# Single-centre real-life observational study on mortality and outcomes: decompressive craniectomy and brain death in traumatic brain injury, haemorrhage, and other cerebral diseases

**DOI:** 10.1007/s00701-024-06170-3

**Published:** 2024-07-06

**Authors:** Isabelle Schröder, Erdem Güresir, Hartmut Vatter, Martin Soehle

**Affiliations:** 1 Department of Anaesthesiology and Intensive Care Medicine, University Hospital Bonn, Bonn, Germany; 2 Department of Neurosurgery, University Hospital Bonn, Bonn, Germany; 3 Klinik für Anästhesiologie und Operative Intensivmedizin, Universitätsklinikum Bonn, Venusberg-Campus 1, 53127 Bonn, Germany

**Keywords:** Decompressive Craniectomy, Intracranial Pressure, Brain Death, Traumatic Brain Injury, Intracerebral Haemorrhage

## Abstract

**Background:**

Decompressive hemicraniectomy (DHC) is used after severe brain damages with elevated, refractory intracranial pressure (ICP). In a non age-restricted population, mortality rates and long-term outcomes following DHC are still unclear. This study’s objectives were to examine both, as well as to identify predictors of unfavourable outcomes.

**Methods:**

We undertook a retrospective observational analysis of patients aged 18 years and older who underwent DHC at the University Hospital of Bonn between 2018 and 2020, due to traumatic brain injury (TBI), haemorrhage, tumours or infections. Patient outcomes were assessed by conducting telephone interviews, utilising questionnaires for modified Rankin Scale (mRS) and extended Glasgow Outcome scale (GOSE). We evaluated the health-related quality of life using the EuroQol (EQ-5D-5L) scale.

**Results:**

A total of 144 patients with a median age of 58.5 years (range: 18 to 85 years) were evaluated. The mortality rate was 67%, with patients passing away at a median of 6.0 days (IQR [1.9–37.6]) after DHC. Favourable outcomes, as assessed by the mRS and GOSE were observed in 10.4% and 6.3% of patients, respectively. Cox regression analysis revealed a 2.0% increase in the mortality risk for every year of age (HR = 1.017; 95% CI [1.01–1.03]; p = 0.004). Uni- and bilateral fixed pupils were associated with a 1.72 (95% CI [1.03–2.87]; p = 0.037) and 3.97 (95% CI [2.44–6.46]; p < 0.001) times higher mortality risk, respectively. ROC-analysis demonstrated that age and pupillary reactivity predicted 6-month mortality with an AUC of 0.77 (95% CI [0.69–0.84]). The only parameter significantly associated with a better quality of life was younger age.

**Conclusions:**

Following DHC, mortality remains substantial, and favourable outcomes occur rarely. Particularly in elderly patients and in the presence of clinical signs of herniation, mortality rates are notably elevated. Hence, the indication for DHC should be set critically.

## Introduction

Untreated brain injuries and diseases often lead to severe disabilities or death. Advances in understanding the pathophysiology, diagnosis, and treatment of these conditions have improved outcomes. This study focuses on one treatment modality, decompressive (hemi)craniectomy (DHC), and examines its effects.

DHC is performed worldwide, often as a last resort for uncontrollable elevated intracranial pressure (ICP). The current procedure involves removing a part of the cranium and the underlying dura mater. The removed bone segment is frozen for potential re-implantation after a certain time interval [25].^.^ Common reasons for performing this surgery include traumatic brain injury (TBI) and space-occupying infarcts of the medial cerebral artery. Other causes, such as intracerebral haemorrhages (ICH), subarachnoid haemorrhages, inflammations, and tumours can also lead to a DHC when ICP becomes uncontrollable [3, 17].

The potential benefits of DHC for patients are highly debated, and identifying factors that can predict the postoperative course is of great significance [2, 3, 10, 14–16, 21, 26]. The mortality rates following DHC vary greatly among different studies and diagnoses. For instance, the range of recorded mortality after DHC for TBI is quite broad, spanning from 19 to 72% across studies like DECRA, RESCUEicp, and a study by Rankothkumbura et al. [2, 3, 10]. In patients with ICH, clear guidelines for performing a DHC are still lacking, with mortality rates reported at 27% three months post-DHC in a study by Esquenazi et al., and 31% after six months in a study by Rasras et al. [5, 22]. Evidence for rarer diagnoses leading to DHC is even scarcer. In Pérez-Bovet’s review, patients with encephalitis exhibited mortality rates around 8% [19], while mortality rates for patients with tumors ranged from 7% (only meningioma) to 66% (various tumor entities) after six months [7, 12].

This study provides a real-life representation of all adult patients, regardless of their age and preoperative characteristics, who presented with one of the aforementioned diagnoses or other rare conditions leading to DHC. Therefore, the study aims to investigate mortality and functional outcomes following DHC, identify predictors of negative outcome and analyse the association with the occurrence of brain death.

## Methods

We retrospectively analysed records from 155 patients, followed by telephone interviews to assess current health status and quality of life.

### Inclusion criteria

Patients undergoing DHC between 2018 and 2020 at the University Hospital of Bonn were analysed. All patients aged 18 years or more receiving DHC for diagnoses including traumatic brain injury (TBI), haemorrhages such as intracerebral (ICH), chronic subdural and rebleeding after surgeries, inflammation, or tumours were included. Patients with DHC after brain infarction or subarachnoid haemorrhage were excluded.

### Retrospective analyses

Patients were identified by their operation and procedure code within the hospital’s patient database. Primary diagnoses were determined, and patients categorised accordingly. Medical records were reviewed and analysed. DHC was defined as primary or secondary depending on whether it was performed within 24 h of injury or thereafter.

### Provision of neurosurgical services

The University Hospital Bonn is a tertiary hospital with a neurosurgical department that provides access to neurosurgical consultant-led care 24 h a day. This includes a dedicated neurointensive care unit, endovascular neuroradiology, inhospital neurorehabilitation unit and helicopter platform within the context of a Level I trauma center.

### Telephone interviews

To assess post-hospitalisation health and to establish a long-term endpoint, telephone interviews were conducted at least 6 months after DHC. Functional outcome and quality of life was classified using questionnaires for modified Rankin Scale (mRS), extended Glasgow Outcome Scale (GOSE), and EuroQol (EQ-5D-5L) [6, 13, 20, 27].

Contact information was obtained from medical records. If patients couldn't be located due to missing contact details, interim patient mortality or other reasons, inquiries were made through local registration offices to update addresses and verify patient status. Patients received written information about the study at least 2 weeks before the interviews. Telephone surveys were conducted with patients when possible; otherwise, family members or caregivers were interviewed.

### Ethics

Ethical approval was granted by the University of Bonn Ethics Committee (Id No. 472/20).

### Statistics

*S*tatistical analysis was conducted using SigmaPlot (Version 14.0, Systat Software GmbH, Erkrath, Germany). Variables were described using mean ± standard deviation in case of normal distribution, and as median as well as interquartile range otherwise. Primary outcome measures included overall mortality (yes/no) and treatment results (favourable/unfavourable). Additional measures included survival time, 6-month mortality, and quality of life.

Patient outcomes were assessed through three approaches. Firstly, functional treatment outcomes were categorised as favourable (mRS 0–3 or GOSE 5–8) or unfavourable (mRS 4–6 or GOSE 1–4). Binary outcomes were analysed by comparing frequencies using chi-squared tests for categorical variables and Student's t-test (for normally distributed values) or Mann–Whitney U-test (for non-normally distributed values) for continuous variables.

Secondary analysis involved evaluating survival time using Kaplan–Meier survival curves and Cox regression analyses. Preoperative variables were individually assessed for their impact on survival time. A combined Cox regression model was created with significant variables.

For 6-month mortality assessment, logistic regression analyses were conducted to identify associated variables. A logistic regression model with significant variables was developed, and a ROC analysis was performed on predictive probabilities.

The significance level was set at α = 0.05.

## Results

**Fig. 1:**
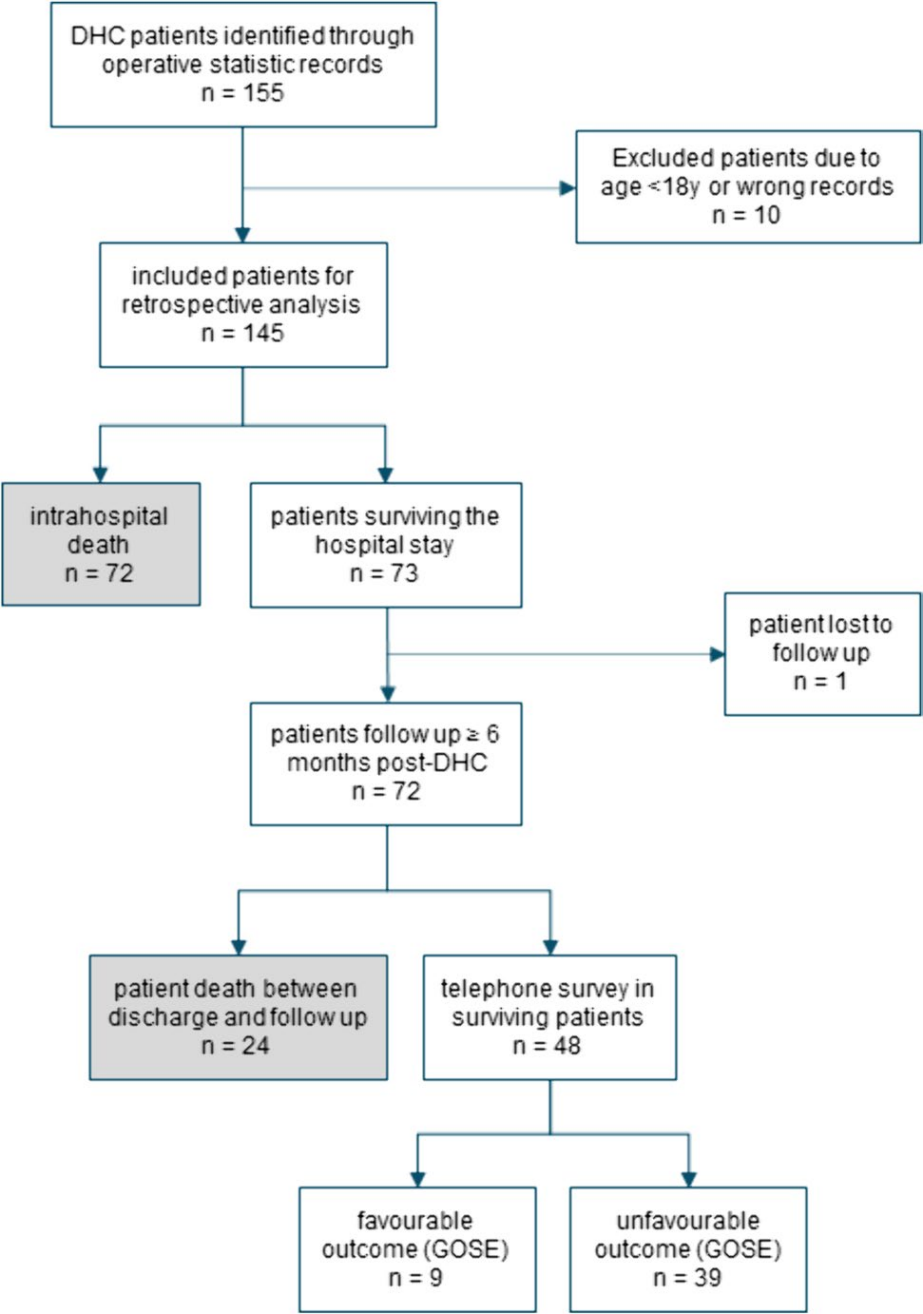
Flowchart illustrating the inclusion and exclusion processes, as well as the patient journey from DHC to follow up

155 patients undergoing DHC were identified, of which 4 surgeries had been classified wrong and 6 were less than 18 years old. One patient was lost on follow-up, leaving 144 patients for final analysis (Fig. 1). The patients showed various diagnoses for undergoing DHC: 70 with TBI, 58 with haemorrhage, 10 with inflammation, and 6 with tumours (Table 1).

**Table 1:** Underlying diseases and timing of decompressive craniectomy in different causes of brain damage

Cause of brain damage	Diagnosis	Number of patients	Number of patients with primary DHC	Number of patients with secondary DHC
Trauma(n = 70)	• mild TBI	11	5	6
	• moderate TBI	5	3	2
	• severe TBI	54	45	9
Haemorrhage (n = 58)	• intracerebral	52	42	10
	• chronic subdural	3	1	2
	• rebleeding after surgery	3	2	1
Inflammation (n = 10)	• meningitis (with (n = 3) or without (n = 4) subdural empyema)	7	0	7
	• brain abscess	2	1	1
	• HIV-associated leukencephalopathy	1	0	1
Tumour (n = 6)	• glioblastoma	2	1	1
	• meningioma	2	1	1
	• metastasis	2	1	1

Primary/secondary DHC: decompressive hemicraniectomy within/after 24 h of injury, TBI: traumatic brain injury

In the cohort, there were 91 (63.2%) male patients and 53 (36.8%) female patients. The mean age of the patients was 56.6 ± 16.8 years, with an age range from 18 to 85 years (Table 2).

**Table 2:** Clinical and demographic characteristics of patients before DHC

	Overall patients (*n* = 144)
Age (years; median [IQR])	59 [44–68]
Sex (male; n (%))	91 (63)
Anticoagulation (n = 110; n (%))	32 (22)
Arterial Hypertension (n (%))	48 (33)
Alcohol or drug abuse (n (%))	27 (19)
Type of admission (n (%))	
Emergency	81 (56)
Transfer	56 (39)
Inpatient	7 (5)
GCS (n = 125; median [IQR])	3 [3-8]
GCS-P (n = 125; median [IQR])	2 [1-8]
Pupillary sign signs of herniation (n (%))	
No pupillary signs of herniation	69 (48)
Anisocoria	38 (26)
Bilateral fixed pupils	37 (26)
Radiologic signs of herniation in CT (n = 143; n (%))	
No radiologic signs of herniation	27 (19)
Midline shift	54 (38)
Herniation	62 (44)
Primary/secondary DHC (primary; n (%))	102 (71)
Time from admission until DHC (h; median [IQR])	5 [2-24]
Time from admission until indication (h; median [IQR])	3 [1-22]
Time from event until DHC (h; median [IQR])	8 [4-27]
Urgency of the surgery (n (%))	
Immediate Intervention	22 (15)
Emergency surgery < 6h	90 (63)
Emergency surgery > 6h	32 (22)

The median time between admission and DHC was 5.0 h (IQR [2.1–24.3]), while the interval between the event and DHC was 7.5 h (IQR [3.8–27.4]). Primary DHC was performed in 102 (71%) patients and 42 (29%) patients underwent a secondary DHC (Table 2).

After a median overall length of stay of 28.3 days (IQR [16.8–43.5]), the 72 (50%) patients who survived were discharged from the university hospital. All patients received inpatient neurorehabilitation.

### Indication for DHC

In 128 out of 144 patients, the indication for DHC was given by pupillary (anisocoria or bilaterally dilated pupils) or radiological signs (midline shift or uncal herniation) of imminent or existing brain herniation. In eight and five patients, DHC was performed because of a progressive deterioration of consciousness or a therapy refractory rise in ICP, respectively. The remaining three patients suffered from a mild or moderate TBI, and the indication for DHC was an expected rapid neurological deterioration due to extensive frontal contusions.

### Mortality

In total, 96 out of 144 patients (66.7%) had passed away by the time of the telephone survey. The study's mortality extended over 21 months, with deaths occurring both intraoperatively and up to 640 days postoperatively. The median time until death was 6.0 days after the DHC (IQR [1.9–37.6]).

During the hospital stay, 72 out of 144 operated patients (50%) died. Among them, 4 (5.6%) had already passed away during the surgery, and 21 (29.2%) within the first 48 h after DHC. Patients who died in the hospital were, on average, 2 years older (age = 58 ± 16 years) than those who survived their hospital stay (age = 56 ± 17 years). The deceased patients were distributed among the following diagnoses: TBI 37 (51%), haemorrhage 27 (38%), inflammation 6 (8%), and tumour 2 (3%). There was no significant difference between the diagnosis groups.

### Brain death

The diagnosis to identify irreversible loss of brain function (brain death) was not conducted routinely for all patients. Brain death was documented in 7 (4.9%) patients who passed away in the hospital. Organ donation occurred in 3 of these patients, representing 3.1% of the overall deceased cases.

### Survival time

A shorter survival time was significantly associated with increasing age, a low GCS, an anisocoria and bilaterally dilated, non-reactive pupils at the time of surgery indication. A trend towards an association with pre-existing anticoagulation could also be observed (Table 3).

**Table 3:** Association of preoperative variables with survival time (Cox-Regression)

Independent Variable	Hazard Ratio	95% confidence interval	Significance (*p*-value)
Age	1.020	1.006 – 1.034	**0.004**
Sex	0.998	0.659 – 1.512	0.994
Anticoagulation	1.415	0.877 – 2.283	0.155
Arterial Hypertension	0.927	0.607 – 1.415	0.725
Alcohol or drug abuse	0.829	0.484 – 1.420	0.495
Type of admission (reference: Emergency)			
Transfer	1.017	0.401 – 2.582	0.971
Inpatient	0.949	0.625 – 1.440	0.804
GCS	0.925	0.873 – 0.979	**0.007**
GCS-P	0.917	0.869 – 0.967	**0.001**
Clinical signs of herniation			
Anisocoria	1.723	1.033 – 2.874	**0.037**
Bilateral fixed pupils	3.974	2.443 – 6.464	**< 0.001**
Signs of herniation in CT			
Midline shift	0.589	0.330 – 1.050	0.073
Herniation	0.959	0.560 – 1.640	0.878
Primary / secondary DHC	0.871	0.558 – 1.361	0.544
Time from admission until DHC	0.999	0.996 – 1.002	0.477
Time from admission until indication	0.999	0.996 – 1.002	0.490
Time from event until DHC	0.999	0.996 – 1.002	0.364
Urgency of the surgery (Reference: Emergency surgery < 6h)			
Immediate Intervention	1.068	0.606 – 1.883	0.821
Emergency surgery > 6h	0.829	0.500 – 1.375	0.468

According to a Cox regression analysis model with age and clinical signs of herniation as covariates, each additional year of age correlated with a 2.0 % increase in the risk of mortality (HR = 1.020; 95% CI [1.006–1.034]; p = 0.004). Anisocoria was associated with a 1.72-fold higher risk of mortality (HR = 1.723; 95% CI [1.033–2.874]; p = 0.037), while bilateral dilated and non-reactive pupils raised the risk by a factor of 3.97 (HR = 3.974; 95% CI [2.443–6.464]; p < 0.001).

### Functional outcome

The surviving patient cohort was contacted by phone a median time of 632 (IQR [348–857]) days post-DHC.

A change in the GOSE classification of patients who initially survived their hospital stay was observed between discharge and telephone survey. Initially, most patients were concentrated in outcome categories 2 and 3. At the time of the telephone survey one-third of patients had passed away (33.3%), while the longer-term survivors showed a distribution toward better outcome categories.

Functional outcomes were assessed for the 48 patients who were still alive and accessible for the telephone survey. The analysis, categorising outcomes as favourable or unfavourable on the mRS or GOSE, showed no association of the functional outcome with any of the preoperative factors.

### Quality of life

Patients and their relatives assessed their subjective health on the EQ-VAS as follows: 3 (6.2%) reported their health at 25% or less, 18 (37.5%) rated their health between 26–50%, 8 (16.7%) between 51–75% and 3 (6.2%) reported greater than 75% health. 16 (33.3%) did not provide feedback.

**Fig. 2:**
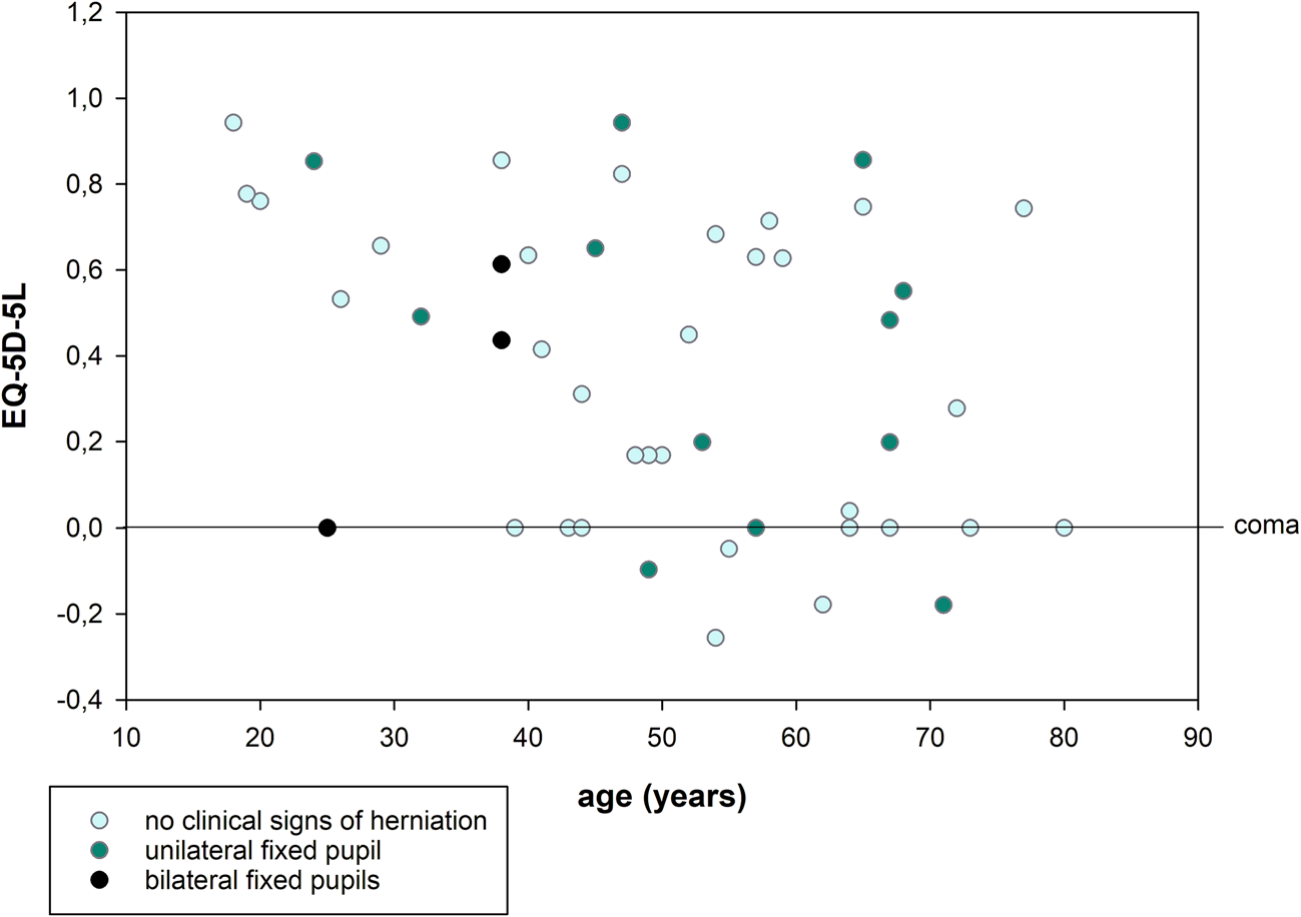
Quality of life assessed by EQ-5D-5L and age; categorized by clinical signs of herniation. Coma patients were assigned a quality of life score of 0 for comparability

At the time of the telephone survey, 10 out of 48 surviving patients (21%) were in a vegetative state. These patients were excluded from the 5Q-5D analysis because no information on pain/discomfort and anxiety/depression dimensions could be obtained (Fig. 2).

### Preoperative Parameters

#### Diagnoses

In terms of patient survival time, there were no significant differences among the four clinical diagnoses (TBI, haemorrhage, tumour, inflammation; p = 0.558).

#### Age

**Fig. 3:**
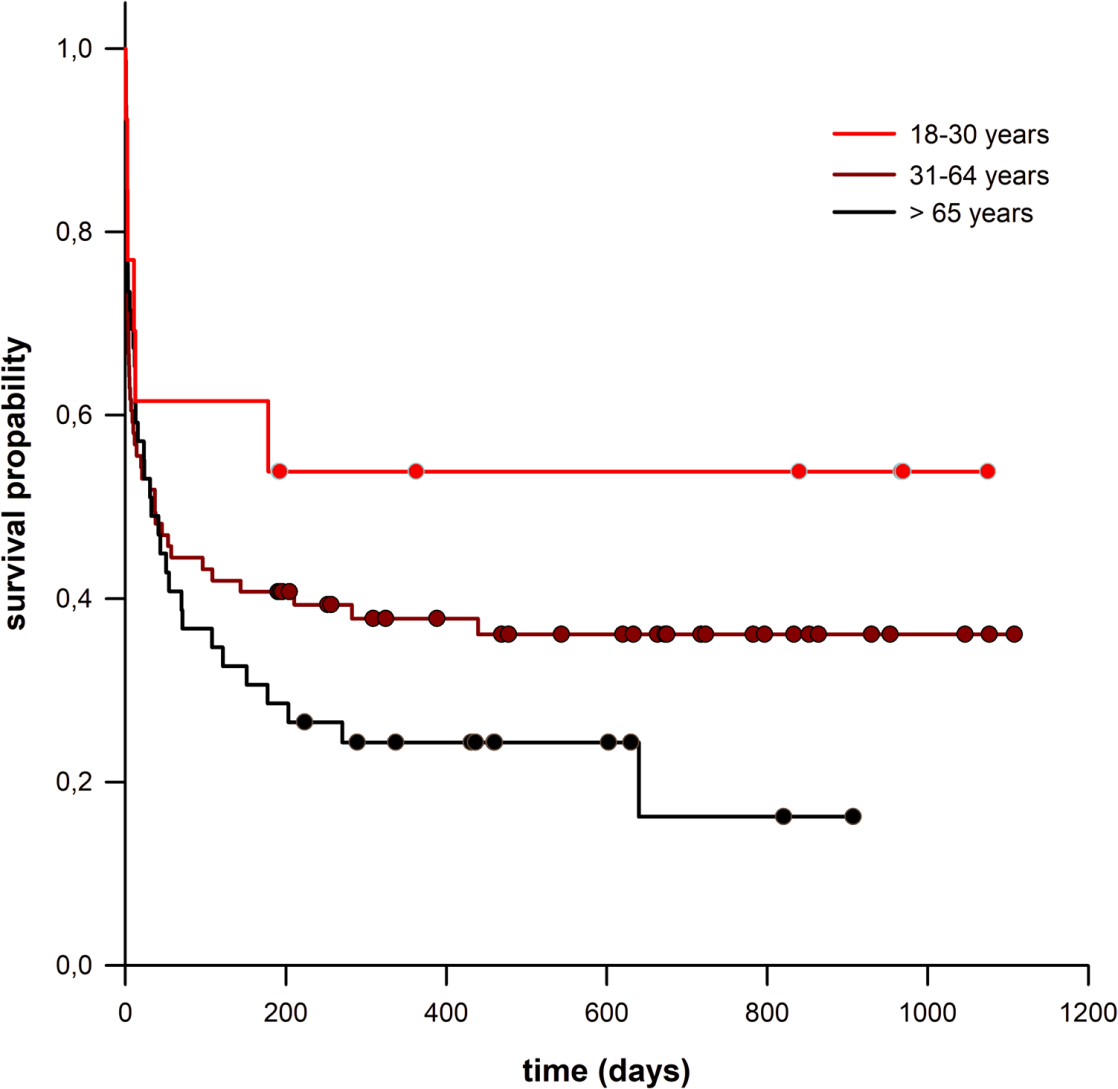
Survival after DHC depending on age categories

Survival after DHC depended on age categories (Fig. 3). A significant age difference was found between deceased (median age: 62 years, IQR [53–73]) and surviving patients (median age: 51 years, IQR [39.5–64]) (p < 0.001). 6-month mortality increased with age (OR = 1.030; p = 0.006), and older age correlated with shorter survival time (HR = 1.020; p = 0.004).

Regarding functional outcomes, a significant association was found between older age of surviving patients and unfavourable outcomes (p = 0.047). Additionally, the assessment of the quality of life was significantly influenced by the patients' age. As age increased, the evaluation of the 5 dimensions of the EQ-5D-5L tended to be less positive (p = 0.018). Concerning subjective health status assessed with VAS, there was no significant correlation with age (p = 0.993).

#### Clinical Signs of Herniation

**Fig. 4:**
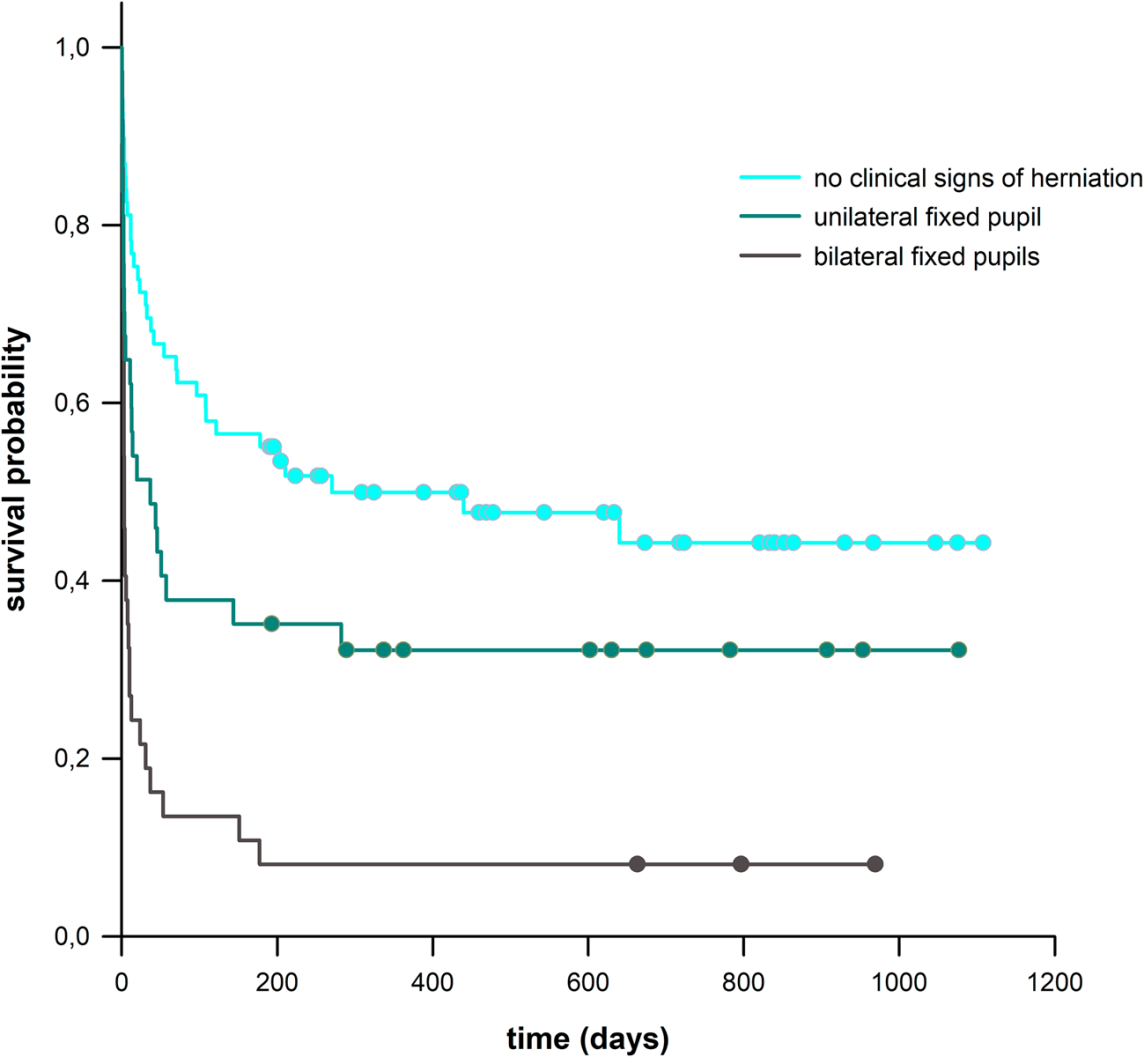
Survival after DHC depending on clinical signs of herniation

Absent pupillary herniation signs, anisocoria, and wide, fixed pupils were observed in 48%, 26% and 26% of patients, respectively. These clinical signs of herniation significantly impacted patients survival time (Fig. 4). A Hazard Ratio of 1.723 (p = 0.037) for anisocoria and 3.974 (p < 0.001) for the presence of bilateral fixed pupils was observed. Six-month mortality was also affected, showing a significant difference between the groups (OR = 3.253; p < 0.001). However, there was no significant association between pupil status and favourable functional outcomes in survivors (p = 0.098). Preoperative pupil status also didn't significantly affect survivors' quality of life (p = 0.946). Notably, only 3 of 37 (8%) patients with initially fixed pupils survived.

In a subgroup of the 37 patients initially presenting with bilateral dilated, fixed pupils, it was found that their ages did not significantly differ from those of all other patients (56.6 years ± 14.7; range = 25–81 vs. 56.6 years ± 17.4; range = 18–85). Furthermore, patients from all pathologies, except for tumours, were represented in this cohort. 23 patients suffered from severe TBI with various bleedings, 10 experienced a hemorrhage and 4 exhibited signs of inflammation (including 2 cases of meningitis, 1 brain abscess, and 1 HIV-associated leukoencephalopathy). Remarkably, only 3 of the patients survived DHC, resulting in a mortality rate of 91.9% within this subgroup. 

#### Other Preoperative Parameters

Pre-existing anticoagulation didn't significantly affect either 6-month mortality (p = 0.072), or survival time (p = 0.155). In contrast, preoperative GCS and GCS-P classification had an impact on survival time (p = 0.007 and p = 0.001) and on 6-month mortality (p = 0.005 and p = 0.001).

Other preoperative variables showed no association with either survival time or 6-month mortality.

The time intervals between the bleeding or trauma event and DHC, as well as between hospital admission and DHC, were not significantly related to survival time and 6-month mortality. Consequently, there was no association between primary or secondary DHC and survival time (p = 0.544).

### Cranioplasty

A cranioplasty (CP) was performed in 45 out of the 48 surviving patients (94%) at a median of 137 days (IQR [120–246]) after the initial surgery.

### Prediction

A logistic regression analysis was conducted to predict 6-month mortality based on significant preoperative factors, including age and clinical signs of herniation. The ROC analysis yielded an AUC of 0.77 (95% CI [0.69–0.84]).

## Discussion

### Mortality

Mortality rates after DHC for various diagnoses are vital in evaluating its treatment efficacy. Different studies examined DHC mortality and compared it to conservative treatment approaches, producing widely varying results (Table 4). For instance, DHC mortality in patients with TBI ranged from 11 to 59% in published studies [1]. This variability partly arises from differences in inclusion/exclusion criteria and limited case numbers. The 6-month mortality in this study was notably higher at 64%. Only one publication reported a higher mortality rate in TBI patients, though their assessment was conducted after 12 months [21].

**Table 4:** Six-months mortality after DHC; Comparison of different studies [2, 3, 5, 7, 10, 12, 19, 21, 22]

	Number of patients with DHC (n)	Median age of patients (years)	6-months mortality (%)
**TBI**			
DECRA [2, 3]	73	24	19
RESCUEicp [10]	201	mean: 32 ± 13	27
Rankothkumbura et al. [21] (12-months mortality)	98	45	72
Our data	70	57	64
**ICH**			
Esquenazi et al. [5] (3-months mortality)	73	52	27
Rasras et al. [22]	13	60	31
Our data	52	64	54
**Inflammation**			
Pérez-Bovet et al. [19] (overall mortality)	37	33	8
Our data	10	52	70
**Tumour**			
Jacobo et al. [12] (overall mortality)	61	mean: 49 ± 14	66
Haq et al. [7] (intrahospital mortality)	14	mean: 46 ± 8	7
Our data	6	mean: 61 ± 11	83

Studies on other diagnoses leading to DHC have also shown lower mortality rates compared to the present study. According to the literature, mortality rates for patients with ICH ranged from 27–31% (in contrast to 54% in this study) [5, 22], for inflammation it stood at 8% (in contrast to 70% here) [19], and for various tumours, the range was 7–66% (versus 83% in our findings) [7, 12]. Potential reasons for these differing mortality rates will be discussed below.

As shown in the study, age is a significant predictor of post-DHC outcomes, with younger patients showing better 6-month survival and lower mortality. However, older patients may have pre-existing conditions and shorter life expectancies, potentially affecting these outcomes. We included patients aged 18 and above, without an upper limit. Some studies, like DECRA [2] and RESCUEicp [10], focused on specific age ranges (15–59 and 10–65 years, respectively), contributing to variations in mortality rates. In comparable studies on inflammation and tumours, the median age of patients was also notably younger [7, 12, 19]. Conversely, in studies involving patients with intracerebral hemorrhage (ICH), the median age was more akin to this one [5, 22].

The clinical signs of herniation determined by pupil status also demonstrated a significant association between the degree of herniation signs (none, anisocoria, or bilateral fixed pupils) and survival time or 6-month mortality. A direct comparison with the DECRA [2] and RESCUEicp studies [10] is not possible as those studies excluded patients with preoperatively wide, fixed pupils. The exclusion in those high-quality studies partially accounts for the overall lower mortality rates observed in comparison to our study. However, it must also be mentioned that the indication for DHC in cases with bilaterally dilated, non-reactive pupils in our hospital is questionable.

Overall, the high mortality can be justified within the context of the often inauspicious underlying condition. In DHC-patients, mortality can be caused by intra- as well as extracranial factors. In this unselected patient group, therapy limitations after the DHC occurred, for example, due to refractory shock, uncontrollable bleeding, or presumed patient wishes.

Furthermore, mortality after DHC must be contextualized with other emergency events that entail similar or even higher mortality rates, such as cardiopulmonary resuscitation. The decision for DHC should therefore be made swiftly in the emergency situation, enabling a prompt determination to potentially limit therapy thereafter.

### Functional outcome

Evaluating post-DHC functional outcomes is crucial to assess the surgery's benefit. Many studies, including this one, use patient interviews with standardised scores such as mRS or GOSE. Categorising outcomes as favourable or unfavourable enhances assessment and comparability but has limited value in reflecting patients' actual well-being.

When comparing studies, significant variations exist in the number of patients with unfavourable outcomes (59% to 80% in the literature vs. 94% in our study). Notably, in the DECRA and RESCUEicp studies [2, 3, 10] and our data, few patients were in the upper GOSE categories at the 6-month assessment (Table 5). Over 95% of patients fell into the lower 6 outcome categories. Depending on the study, 8.5% to 12% of patients were in a vegetative state (GOSE 2) at this point. At 12 months [2, 3, 10] and 36 months [21], more patients improved to higher outcome categories, but mortality also increased. In summary, patients tended to shift toward extreme outcome categories over time.

**Table 5:** Comparison of DHC outcomes in TBI patients. Unfavourable outcomes correspond to GOSE 1-4, and death is classified as GOSE 1 [2, 3, 10, 21]

	DECRA [2, 3]		RESCUEicp [10]		Rankothkumbura et al. [21]		Our data
**Timing of follow up [month]**	6	12	6	12	12	36	≥ 6
**Number of patients (n**)	73	73	201	194	89	89	144
**GOSE (%):**							
1	19	21	26.9	30.4	71.9	73.0	66.7
2	12	11	8.5	6.2	0	0	9.0
3	25	19	21.9	18.0	3.4	0	11.8
4	14	8	15.4	13.4	4.5	2.3	6.3
5	18	14	10.0	10.3	3.4	5.6	0.7
6	8	19	13.4	11.9	3.4	0	2.1
7	3	5	2.5	7.2	5.6	10.1	3.5
8	1	3	1.5	2.6	7.9	9.0	0
**Outcome:**							
Unfavourable [%]	70	59	72.7	68.0	79.8	75.3	93.8
Favourable [%]	30	41	27.4	32.0	20.2	24.7	6.3

This study was comparable to Rankothkumbura’s in terms of a significant association between age and functional outcome (p = 0.012 vs p < 0.001) [21].

Among survivors, there was no significant difference in preoperative signs of herniation between those with favourable and unfavourable outcomes. Rankothkumbura et al. found that good preoperative pupil reactivity was linked to better functional outcomes at one and three years, while pupil symmetry had no impact [21]. Notably, although not statistically significant due to the small sample size, none of the three surviving patients with preoperative non-reactive pupils in our data showed a favourable outcome. One of these patients had a vegetative state and represented the poorest outcome among young patients (< 30 years) in the cohort.

### Quality of life

Comparing our patient cohort's quality of life was challenging because many well-known studies [2, 3, 10] primarily focused on functional outcomes rather than subjective quality of life. We compared our EuroQol questionnaire-based quality of life assessment to similar studies. However, direct EQ-5D value comparisons were challenging due to differences in country-specific analysis models. Furthermore, published studies didn't evaluate comatose patients, and we had to exclude those patients from our analysis due to their inability to participate. Comparing the EQ VAS was simpler as it directly assesses subjective health, without complex international models. Nonetheless, when comparing these values, it's important to take into account the impact of cultural and individual factors on this perception. Notably, Malmivaraa et al. reported that their patients with DHC after TBI rated their health significantly better than ours [15, 16].

Increasing age showed a negative correlation with the quality of life (EQ-5D-5L), likely also influenced by pre-existing health conditions.

### Diagnoses

Various diagnoses leading to DHC have differing levels of research support, making the indication for this procedure challenging. Overall, the surgery's benefit is questioned, given the trade-off between reducing mortality and increasing postoperative morbidity [14, 26]. For the diagnosis of ICH, the performance of DHC is not yet evidence-based due to limited research results. Even though there is more research on TBI [2, 3, 10], a clear recommendation for DHC's indication and execution remains elusive.

Presently, German guidelines recommend individualised DHC decisions after discussions with the patient's family regarding the patient's wishes [11]. Few studies have explored DHC for inflammatory and tumour-related conditions, leading to a lack of clear indications. In our study, no significant differences in mortality and functional outcomes were found among the different diagnoses.

### Inclusion criteria

Unlike published randomised control trials, this study is a real-life study that entails a broad and diverse patient population. Patients aged ≥ 18 were included, regardless of various baseline criteria, including pupillary status, anticoagulation, and Glasgow Coma Scale (GCS).

Larger studies such as DECRA [2] and RESCUEicp [10] reported better outcomes and lower mortality rates than this study. These differences may be attributed to variations in exclusion and indication criteria, with age and baseline conditions playing a significant role in functional outcomes.

### Timing of DHC

Studies found that DHC before herniation signs or within 48 h of infarction reduces mortality and improves results, while benefits are unclear for DHC after signs of herniation or beyond 96 h post-infarction [9, 24]. Timing of DHC in TBI is debated. Arguably, adults benefit more from DHC within 24 h of the injury, while children benefit from DHC regardless of timing [24].

Our study examined preoperative timeframes and found no significant correlations with patient outcomes or mortality. Categorising primary and secondary DHC by surgery timing within 24 h or later also showed no significant outcome differences.

### Brain death

In this retrospective study, limited data prevented us from making strong conclusions about the link between DHC and brain death. However, the occurrence of complete brain death in 7 patients after DHC challenges the common belief that DHC prevents brain death. This suggests that even after DHC in patients with primary brain injuries, an increase in ICP can still occur, potentially leading to a brain death due to decreased cerebral perfusion pressure (CPP) and progressive cerebral hypoperfusion [18, 23].

All 7 patients suffering brain death in our study had preoperative signs of brain herniation either clinically or through imaging, indicating that surgical decompression could not undo pre-existing herniation in these cases. However, based on the data in this study, it is not possible to determine whether brain death can occur after DHC in patients with preoperatively elevated ICP without or with early signs of herniation.

### Neurotrauma care standards

Dasic et al. [4] reviewed the characteristics of neurotrauma services in major trauma centers and identified a matrix for unmet research needs in neurotrauma. Several aspects discussed in our study align with this matrix, specifically in areas such as prognostication, service provision, clinical/surgical best practices and follow up [4]. By integrating the findings and recommendations from their scoping review, our work enhances the narrative on the indications and management of patients undergoing DHC. This alignment emphasises the relevance and applicability of the study's insights within the framework established by Dasic et al., ultimately contributing to the advancement of neurotrauma care standards [4].

### Limitations

The retrospective design of this study and resulting data limitations affected its reliability. Limited direct patient contact and reliance on information from caregivers also influenced results. Analysing functional scores, especially with a limited number of patients having favourable outcomes (15 on mRS, 9 on GOSE), posed challenges. Of the preoperative variables, only age significantly correlated with survivors' functional outcomes probably due to the small sample size. Another limitation was the artificial division of results into favourable / unfavourable outcomes, commonly used but not objectively defined. Determining what constitutes a worthwhile life is subjective, varying across cultures, families, and individuals. These considerations are more ethical than medical, lacking a clear-cut answer [8].

The broad range of indications for patients with very different diagnoses and prognoses was also a limitation of our study. All patients were operated on at a life-threatening stage of their disease, which makes the courses of the disease more comparable. However, the sometimes small number of cases in individual diagnostic groups must also be seen as a limiting factor.

Furthermore, data analysis was hindered by the timing of patient follow-ups, which occurred between 6 months and 3 years post-DHC without standardisation to a specific time point.

## Conclusion

The findings support the scepticism surrounding decompressive (hemi)craniectomy for managing elevated intracranial pressure. Mortality rates are high (67%) across all diagnoses, particularly in patients with severe preoperative conditions. Full rehabilitation is rare, with slightly better prospects in younger patients without preoperative herniation signs. The observed trends for age and herniation sign in this study align with existing literature, emphasising the need for additional research to establish clear guidelines and criteria. Overall, DHC managed to save individual patients with an acceptable quality of life, but brain death situations could not be prevented. The role of DHC can be considered more as a measure to gain time and achieve clarity on individual prognosis and potential treatment goals.

## Data Availability

The data that support the findings of this study are not openly available due to reasons of sensitivity and are available from the corresponding author upon reasonable request. Data are located in controlled access data storage at University Hospital Bonn.
